# Phenological response to temperature variability and orography in Central Italy

**DOI:** 10.1007/s00484-021-02190-1

**Published:** 2021-11-30

**Authors:** P. B. Cerlini, M. Saraceni, F. Orlandi, L. Silvestri, M. Fornaciari

**Affiliations:** 1 Centro Interuniversitario di Ricerca sull’Inquinamento e sull’Ambiente Mauro Felli (CIRIAF) - Centro di Ricerca sul Clima e Cambiamenti Climatici (CRC), University of Perugia, Perugia, PG Italy; 2 Dipartimento di Ingegneria Civile e Ambientale (DICA) - Centro di Ricerca sul Clima e Cambiamenti Climatici (CRC), University of Perugia, Perugia, PG Italy

**Keywords:** Phenology, Meteorology, Willow, Orography

## Abstract

Even if the sensitivity of vegetation phenology to climate change has been accepted on global and continental scales, the correlation between global warming and phenotypic variability shows a modulated answer depending on altitude, latitude, and the local seasonal thermal trend. To connect global patterns of change with local effects, we investigated the impact of the observed signal of warming found in Central Italy on two different willow species, Salix acutifolia and Salix smithiana, growing in three phenological gardens of the International Phenological Gardens’ network (IPG) located in different orographic positions. The time series of temperatures and phenological data for the period 2005–2018 were analysed first to find trends over time in the three gardens and then to correlate the recent local warming and the change in the two species phenology. The results confirmed the correlation between phenological trends and local trend of temperatures. In particular: budburst showed a trend of advancement of 1.4 days/year on average in all three gardens; flowering showed a divergent pattern between the gardens of either advancement of 1.0 days/year on average or delay of 1.1 days/year on average; while senescence showed a delay reaching even 3.3 days/year, although significant in only two gardens for both species. These trends were found to be correlated mainly with the temperatures of the months preceding the occurrence of the phase, with a shift in terms of days of the year (DOY) of the two species. Our conclusion is that the observed warming in Central Italy played a key role in controlling the phenophases occurrences of the two willow species, and that the orographic forcing leads to the different shift in DOY of phenophases (from 5 to 20 days) due to the local thermal forcing of the three phenological gardens.

## Introduction

The link between plants life cycle and the warming of the environment in which they are growing has been established in the literature for large-scale and continental data (White et al. 1997; Zhang et al. 2007; Visser and Both 2005; Lee et al. 2018; Piao et al. 2019) for many species (Cook et al. 2012; Wolf et al. 2017; Seyednasrollah et al. 2020) and specific regions with a strong influence of orographic forcing (Gutierrez et al. 2009; Ziello et al. 2009). Generally, the relationship between global temperature record increase and change in the timing of plant life cycle can be seen as a cause-and-effect relationship (Visser and Both 2005; Zheng et al. 2006). Indeed, there is a consensus on the occurrence of earlier budburst and flowering in spring and delayed autumn leaf phases across Europe, Asia, and North America in the last decades as a consequence of global warming (Gordo and Sanz 2010). However, if climate change has affected the phenology of species in general terms (Badeck et al. 2004; Orlandi et al. 2016; Richardson et al. 2013), it remains difficult to interpret these global shifts in terms of local and climatic variability of temperatures. This is due to the specificity of factors measuring the local effect and its consistency with the global trend of variability among species and even among populations within species.

In particular, the timing of species phenology is affected by global warming and the climate feedbacks connected to it, including the differential raising of temperatures due to orographic gradients. As a matter of fact, the lapse rate of temperature per kilometer is modified by the warming with elevation. This is changing the surface temperature above mountainous reliefs shifting the seasonal and annual temperature cycle, in a way that is depending on the local trend of warming, for instance, because of the complex orography. On the other hand, the variation of the phenological traits induced by different temperature and solar radiation regimes at various elevations has a deep impact on tree fitness. Due to the strong relationship between phenology and fitness, plants subjected to different environmental conditions will undergo changed physiological inductions for leaf opening and senescence dates (Worrall 1983; Howe et al. 2003; Ohsawa and Ide 2008).

High-elevation environments impose severe limitations on phenological traits, and consequently, plants of the same species may show a wide range of morphological and physiological variation along latitudinal gradients (Oleksyn et al. 1998; Körner 2003), particularly in leaf phenology (Vitasse et al. 2009).

Contradictory results have been reported about leaf senescence significant cline, where some plant species at low elevations have started to senesce earlier than those from high elevations, but others species have presented an opposite tendency showing small amount of evidence of leaf senescence among natural tree populations, particularly along altitudinal gradients (Deans and Harvey 1995; Chmura 2006). One of the main environmental effects at high elevation is the lowering of temperatures at the end of the growing season, which may cause early leaf drop, while many authors report that the growing season responds to latitude due to variations in day length. High latitude populations generally begin to senesce or to finish their growth earlier than low latitude populations (Hänninen et al. 1990; Deans and Harvey 1995; Mimura and Aitken 2007; Jensen and Hansen 2008).

Therefore, the shift in phenological phases may be related to elevation in a non-linear way (Rafferty et al. 2020) and it may be seen across different species as a key for analyzing them, as done for Italian forests in (Bajocco et al. 2019), where the phenology showed clear elevation gradient.

For the Mediterranean region, the asseverated trend of temperature increase, reported by the IPCC (Pachauri et al. 2014) has been linked to the change in plant life especially for vineyard (Ruml et al. 2016; Fraga et al. 2016) and olive trees (Orlandi et al. 2014). Linked to these temperature trends and their influence on phenology, many studies have been carried out in northern Italy (Tomasi et al. 2011) considering also the effect of elevation on plants (Ziello et al. 2009; Pellerin et al. 2012), while in Central Italy, the influence of raising temperature records on vegetation and agriculture has been shown (Todisco and Vergni 2008).

Then since the climatic data set over the Umbria region, characterized by a very complex orography, has shown a strong effect of warming at the surface in the last decade (Cerlini and Silvestri 2019), one may ask to what degree the climate warming has changed the phenological phase timing in this region and if this has had a different effect on populations of a given species across different elevations during this period.

To answer these questions, we have selected two different willow species, *Salix acutifolia* Willd. and *Salix smithiana* Willd., growing inside three “phenological gardens” located in Central Italy, in the Umbria region, and the neighboring region of Lazio. Both regions are mountainous and located at mid-latitudes in a continental area with a temperate climate. This has been done to consider two different species of the same shrub and for these it has been possible to obtain a long series of congruent data from the three gardens, spanning from 2005 to 2018. These willow species were the first ones to be cloned by the “International Phenological Gardens of Europe” network (IPG) and spread in Italian phenological gardens from early nineties, representing the link with the same IPG network (http://ipg.hu-berlin.de). The impact of the orographic forcing over the climate at a regional scale, and the elevation difference among the phenology data set used, may change the global shift of phenology phases during the considered period.

Therefore, the study primarily focuses on assessing existing phenological trends in the region, particularly for budburst, flowering, and leaf senescence for the two species, extending the study already done on the same species (Orlandi et al. 2020).

Secondly, the research examines the signals of increasing temperatures in the region by calculating the trends of yearly, seasonal, and monthly temperatures in the three gardens. Indeed the chosen period, from 2005 to 2018, includes the last 10 years, where the global increase of temperatures has been accepted as a strong climatic signal by the last IPCC reports (Pachauri et al. 2014; Allen et al. 2019) and it has been reflected by climate indices connected to plants computed over the region (Cerlini and Silvestri 2019).

Finally, the correlation between the above said phenology phases and temperatures data trends have been analyzed. The basic idea has been to find a temperature signal that properly correlate both species for all three sites, to find common patterns, given the differences between the species and their specific location. The aim is to measure the impact that both temperature trends and local climatic features due to the complex orography have on phenotypic variation, thus on contrasting patterns of observations among the two species. Through this, it will be possible to link the consistency of behaviours among gardens and species, trying to reconcile the local variability with global trends.

## Material and methods

### Phenological data

**Fig. 1:**
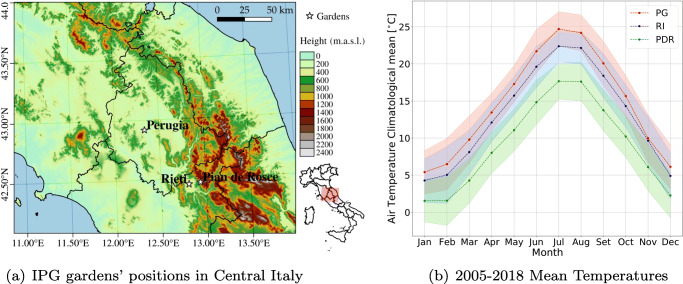
Central Italy map, with the three phenological gardens position and their temperature climatology mean for the period 2005–2018. The garden of Perugia (PG) is in red, Rieti (RI) is in blue, and Pian de Rosce (PDR) is in green

The phenological records used in this study come from three phenological gardens located in central Italy at different altitudes and terrains. One is in the Umbria region, in the province of Perugia, and the other two are in the Lazio region, in the province of Rieti, as shown in Fig. 1(a).

The garden with the lowest elevation is the one in Perugia (PG). It is located in the Umbrian countryside (latitude, 43^∘^ 00’ 40” N; longitude, 12^∘^ 14’ 52”), positioned on the south/south-east slope of the hill, with an elevation between 260 m a.s.l. and 270 m a.s.l.. The other two in order of height are the gardens of Rieti (RI) and Pian de Rosce (PDR). The first is located in the plain of the Rieti basin, surrounded by mountains (latitude: N 42^∘^ 25’ 30”; longitude: E 12^∘^ 49’ 45”), 5 km from Rieti at a height of about 380 m, while the second is located on one of the basin’s mountain, Mount Terminillo (latitude: N 42^∘^ 28’ 40”; longitude: E 12^∘^ 56’ 28”), 15 km from Rieti at a height of 1050 m. The latter is positioned on a west-facing plain zone well below the summit of the mountain.

The main philosophy behind the IPG garden management is based on the fact that all plants are biological indicators that must be allowed to grow as naturally and as long as possible being in this manner influenced only by the natural site-specific environmental variables (Orlandi et al. 2016). The plant species were obtained from mother plants given by the German Weather Service, which is the European coordinator for the distribution of IPG clones. The main objective of the IPGs is the large-scale (European) comparison of climate and weather influences on the growth of gardens’ plants for studying the environmental influence on vegetatively propagated plants from the same mother plant. The two willow species considered in the study, *Salix acutifolia* Willd. and *Salix smithiana* Willd., are caducous shrubs belonging to the Salicaceae family. They usually flower between February and March, before they flush their leaves and they fructify by the end of spring, between May and June. The willow trees, among the other plants’ indicators, were planted in the mid-nineties in the Perugia garden, which is one of the oldest Italian ones, and in the early two-thousands in the other two gardens. Thus, the common recording period for the three gardens under study is 14 years from 2005 to 2018. The chosen period allows comparing the plant’s responses to elevation and climate. The four phenological events recorded in the garden, according to the BBCH international scale (Saska and Kuzovkina 2010), are defined as:

- BBCH11: First leaves unfolded, budburst;
- BBCH65: Full flowering: 50*%* of flowers open, first petals may be fallen;
- BBCH95: 50*%* of leaves fallen;
- Leaf Period: number of days between leaf unfolding and leaf falling for the same species during the same year at the same locality, thus BBCH95 - BBCH11.

These events were estimated as Day Of the Year (DOY hereafter) every year through weekly phenological observations realized directly in the gardens. The observations in each garden were conducted on three individuals of each willow species. Eventually, the mean date of the onset of each phenophase was calculated as an average of the three plants. This use of population monitoring was done to limit the variability of the single plant.

### Climate data

To analyze the climate and the recent warming in the garden areas and to assess the influence of warming in central Italy on the life cycle of the willow trees through statistical correlation, some of the indices recommended by the WMO Commission for Climatology (Zhang et al. 2011) have been computed. These are yearly seasonal and monthly maximum, minimum, and average temperatures. The temperature time series belonging to the meteorological dataset of the Umbrian agro-meteorological network have been analyzed. The latter is made of a combination of different smaller stations networks providing mainly precipitation and temperature observations. The network temperature data have been recently validated using the WMO (World Meteorological Organization) standards (Cerlini et al. 2020). The chosen stations were the nearest meteorological stations to each of the three phenological gardens. This has been done considering the meteorological stations representative of the nearby area, after the process of data validation. In the case of the Perugia garden, the daily mean, maximum, and minimum temperature data were provided by the nearby station, about 50 m from the plant site, belonging to the Umbrian network and also to the Italian Agrometeorological Network. As for the gardens of Rieti and Pian de Rosce, the daily mean, maximum, and minimum temperature data come from the closest stations belonging to the “Apennines centre of Terminillo Mountain” [CAT], managed by the University of Perugia and integrated within the Umbrian network, as stations in regions bordering Umbria.

### Methods

First of all, a statistical analysis of the phenological phases and the temperature indices annual variation for each site has been carried out through the linear regression method and Pearson’s correlation. While it is globally accepted that temperature and its indices (in our case maximum, minimum, and average temperatures) follow the normal distribution and can be studied via linear regression (Klein Tank et al. 2009), to check whether the phenological data follow a normal distribution, the histograms of the phenological data have been plotted (not shown) and the Shapiro Wilks test has been carried out, to confirm or reject the normal distribution hypothesis. This test has proven to be more powerful than other normality tests when it comes to lower sample size (Razali et al. 2011). The result of the test was that the phenological data of the three phases for the two species in the three gardens do not differ from the Gaussian distribution at a significance level of 5*%*, thus indicating that linear regression can be used to study trends for these data sets. Moreover, this type of analysis has been applied in the past (Guo et al. 2015; Fu et al. 2014), and also for time series of similar size to the case under analysis (Pudas et al. 2008).

The temperature analysis focuses not only on the annual variation of the yearly maximum, minimum, and average temperatures but also on the annual variation of monthly and seasonal temperatures, smaller periods during which the onset of a particular phenophase could have been mostly affected by temperature change. For what it concerns this temperatures’ annual variation, the analysis suggested by the WMO Guidelines (Klein Tank et al. 2009) is using the linear regression, given its sensitivity to outlying observations near the beginning or the end of the available data record, with the use of the Mann Kendall’s non-parametric test (MK test) (Mann 1945; Kendall 1975). The modified version of the test was used to account for the serial correlation of the recorded temperature data (Yue and Wang 2004).

Finally, to relate phenological phases with temperature indices, least squared linear regression has been used, given the effectiveness in previous studies (Menzel et al. 2006; Ruml et al. 2016; Fraga et al. 2016), together with the Pearson’s correlation coefficient. This was done in the aforementioned period when data were available for all three gardens, i.e. from 2005 to 2018. The statistical correlation was performed using a 3-year running mean on phenological data. This was done to reduce the uncertainty associated with the phenophase data. Although the analyzed data derive from an average of three plants in the gardens, they are still very noisy data, with approximately one week error to it. The moving average has been done for smoothing these fluctuations, thus reducing this error.

The statistical analysis is performed using Python programming language and the specific python package for Mann Kendall test (Hussain and Mahmud 2019).

## Results

### Observed phenological phases trends

**Fig. 2:**
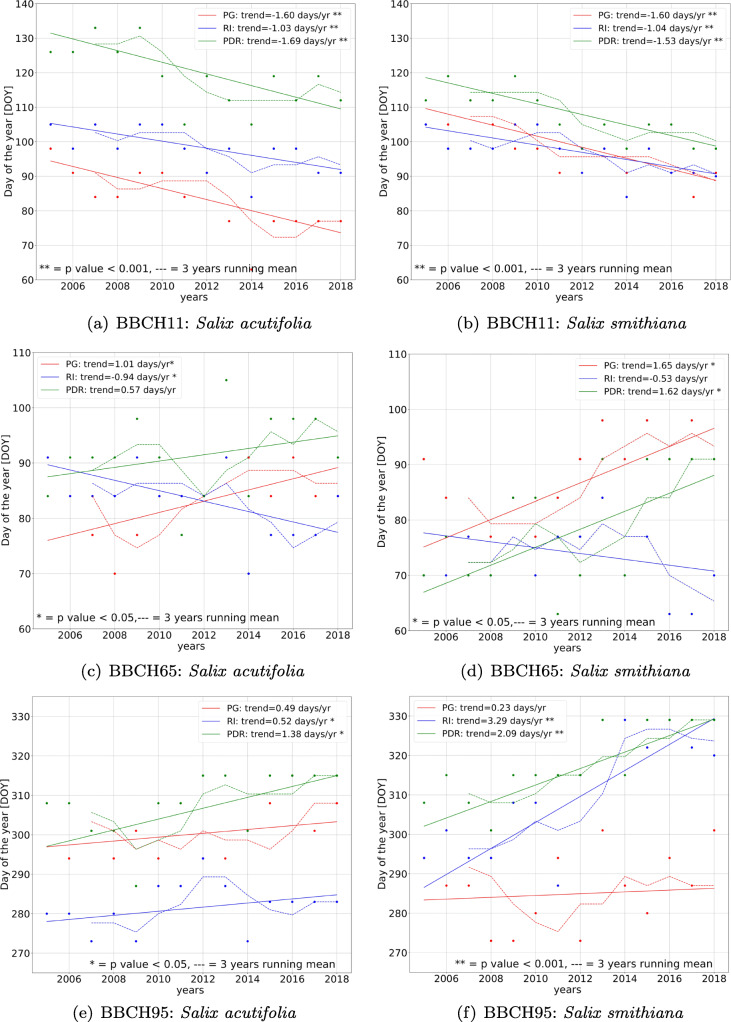
Phenological phases BBCH11, BBCH65 and BBCH95 trends for both *smithiana* and *acutifolia* in the 14-year period. Both the original time series (continuous line) and the running mean of 3 years (dotted line) are plotted in the graphs, in red for the garden of Perugia, in blue for Rieti and in green for Pian de Rosce. The significance is in most cases of *p*< 0.05. In some cases the *p* value is *p* < 0.001

Linear regression analysis shows the general presence of significant trends in the three gardens for both species (Table 1). Starting with budburst (BBCH11), it is the phenological phase for which both species of each garden show significant trends of yearly advancement over the past 14 years (*p* < 0.05^1^) in time of between 1 and 2 days. This means that from the beginning/half of April, the leaf flushing advanced to the middle/end of March in general. Regarding plant’s flowering (BBCH65), what is immediately noticeable is the different behavior of both species, *Salix acutifolia* (Fig. 2(c)) and *Salix smithiana* (Fig. 2(d)) among the gardens of Perugia and Pian de Rosce, and the garden of Rieti. While in the former two there is a positive trend over time, meaning a delay of the plants in realising the flowers from 0,5 to 1.6 days/year, in that of Rieti, an advance is noted of 0.5 to 1 days/year.

**Table 1:** Phenological phases trend analysis for the three years running mean values

Phenophase	Species	Garden	m ± *σ*	Trend(d/y)	*R*^2^
BBCH11	*Salix acutifolia*	PG	82.4 ± 1.9	–1.60**	–0.84
		RI	97.6 ± 1.3	–1.03**	–0.83
		PDR	118.8 ± 2.14	–1.69**	–0.82
	*Salix smithiana*	PG	97.6 ± 1.8	–1.60**	–0.94
		RI	96.4 ± 1.3	–1.04*	–0.83
		PDR	107.1 ± 1.8	–1.53**	–0.90
BBCH65	*Salix acutifolia*	PG	83.6 ± 1.4	1.01*	0.73
		RI	82.6 ± 1.2	–0.94*	–0.81
		PDR	91.7 ± 1.1	0.57	0.52
	*Salix smithiana*	PG	87.5 ± 1.8	1.65*	0.87
		RI	73.7 ± 1.2	0.53 NS	–0.44
		PDR	79.1 ± 1.9	1.62*	0.85
BBCH95	*Salix acutifolia*	PG	300.6 ± 2.1	0.49	0.40
		RI	281.9 ± 1.3	0.52*	0.45
		PDR	307.4 ± 1.8	1.38*	0.78
	*Salix smithiana*	PG	285.1 ± 1.5	0.23	0.2
		RI	311.2 ± 3.7	3.29**	0.92
		PDR	317.7 ± 2.2	2.09**	0.97
LP	*Salix acutifolia*	PG	217.6 ± 2.4	1.97*	0.86
		RI	185.2 ± 2.2	1.49*	0.70
		PDR	188.6 ± 3.8	3.07*	0.85
	*Salix smithiana*	PG	187.4 ± 2.2	1.83*	0.87
		RI	215 ± 5	4.35**	0.92
		PDR	210.6 ± 3.8	3.61**	0.96

* *p* < 0.01, ** *p* < 0.001

Bold values mean a significance of *p* < 0.05

Finally, with regard to leaves senescence (BBCH95) in the garden of Perugia, a smaller annual variation for both *Salix acutifolia* and *Salix smithiana* can be noticed (see Fig. 2(e) and (f)), that is not particularly marked and generally not significant (Table 1). In the other two gardens, the two species show positive significant trends of leaf senescence’s delay from 0.5 to 3.29 days/year for both species (Table 1).

In general, where the trend is significant, the delay in leaf senescence means an extension of 10 to 20 days more of the leaf period.

Between the three gardens, apart from the magnitude and the presence of phenological trends, there is a difference in terms of DOY due to their orographic positioning. For the budburst there is a clear scaling with elevation for *Salix acutifolia* (Fig. 2(a)) translating to the fact that in the garden in the highest position the event occurs on average 10–25 days later, compared to the gardens at lower elevation. For *Salix smithiana* (Fig. 2b) instead, the scaling still holds but is less marked because in this case, the plants in the two gardens of Perugia and Rieti tend to uniform in the occurrence of the phase. In general, in the garden at a lower elevation, the leaves are released earlier.

In the case of flowering, the difference in the positioning of the gardens leads to a delay of 15 days on average in its occurrence in Pian de Rosce compared to the lower gardens, only in the case of *Salix acutifolia*. (Table 1). Instead, in the case of *Salix smithiana*, the Pian de Rosce garden seems to anticipate that of Perugia by about 5 days on average.

In general, it seems that over time, the trends of the phenological phase of flowering and budburst are leading the two phenophases to overlap (Fig. 2 for BBCH65 and BBCH11).

Regarding senescence, it is noticeable that in the garden of Pian de Rosce the plants senescence occurs after it does in the other two gardens (Fig. 2). Indeed, by looking at the mean values, senescence occurs around the beginning/half of November for Pian de Rosce, while it occurs around the half/end of October in Perugia and Rieti. However, this shift due to the elevation decreases significantly especially in the most recent years, where the senescence events of the garden of Rieti for *Salix smithiana*, or Perugia for *Salix acutifolia* tend to coincide with those of Pian de Rosce. The elevation factor does not seem to influence the senescence trends for the lower gardens.

In general, the combination of the senescence delay and the budburst advancement produces a significant leaf Period (LP) lengthening trend in the three gardens for the two species that is about 20 days on average in the last 14 years (Table 1).

### Observed temperature trends

**Fig. 3:**
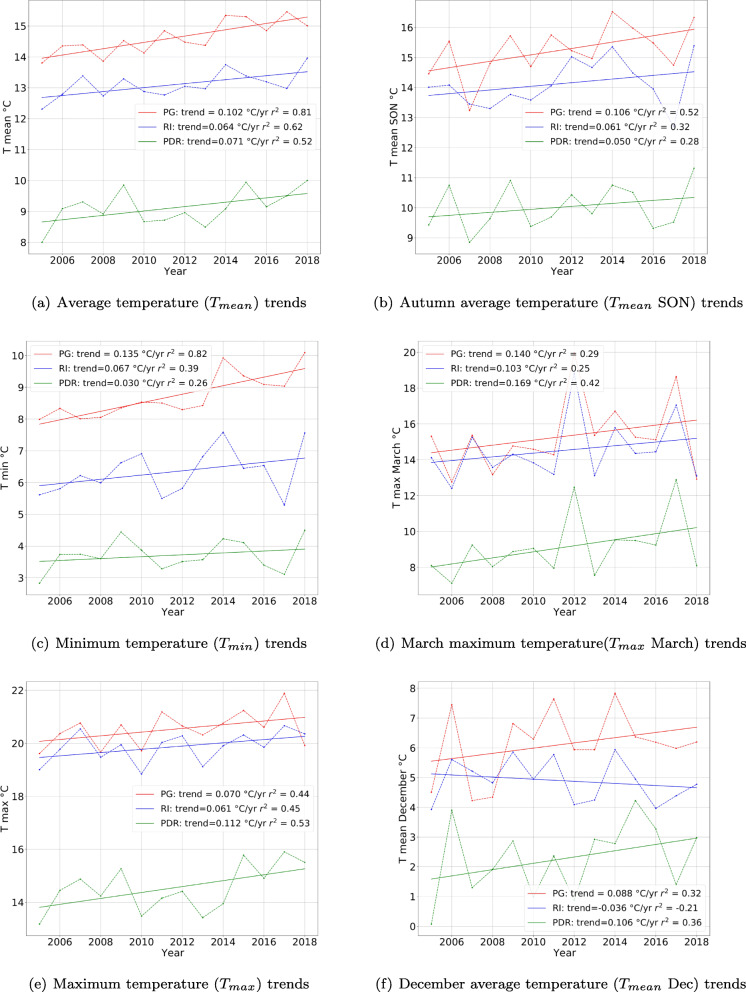
Temperature trends for the annual average temperature Fig. 3(a), maximum temperature in Fig. 3(e), minimum temperature in Fig. 3(c) and for the most significantly correlated indices, March maximum temperature in Fig. 3(d), December average temperature in Fig. 3(f) and Autumn (September-October-November) SON average temperature in Fig. 3(b) for Perugia (PG) in red, Rieti (RI) in blue, and Pian de Rosce (PDR) in green

The gardens where the two willow species grow, show a general increase in temperature over the last 14 years. The average (*T*_*mean*_) in Fig. 3(a), the minimum (*T*_*min*_) in Fig. 3(e) and the maximum temperature (*T*_*max*_) in Fig. 3(c) present positive trends in the three gardens sites. The trends, for *T*_*mean*_ and *T*_*min*_, are more marked in the Perugia site than in the Rieti and Pian de Rosce sites (Table 2). While in Perugia *T*_*mean*_ is significantly increasing by 0.10^∘^*Cyr*^− 1^, in Rieti and Pian de Rosce the increase is lower (Table 2). As for *T*_*max*_ trends, the more marked one is for the Pian de Rosce site with the highest increase of 0.11 ^∘^*Cyr*^− 1^.

**Table 2:** Climate indices analysis

Index (^∘^*C*)	Garden	m ± *σ*	Trend (^∘^*Cyr*^− 1^)	MK Trend (z)
*T*_*mean*_ (average temperature)	PG	14.6 ± 2.0	0.10**	0.10** (10.8)
	RI	13.05 ± 1.93	**0.06**	0.06** (6.9)
	PDR	9.07 ± 1.74	**0.07**	0.08**(3.9)
*T*_*min*_ (minimum temperature)	PG	8.7 ± 1.6	0.13**	0.11**(6.3)
	RI	6.2 ± 1.6	0.07	0.08** (3.5)
	PDR	3.7 ± 1.4	0.03	no trend 0.03 (0.64)
*T*_*max*_ (maximum temperature)	PG	20.5 ± 2.4	0.07	0.09** (4.4)
	RI	19.8± 2.2	0.06	0.07** (4.6)
	PDR	14.7 ± 2.0	**0.11**	0.12**(3.3)
*T*_*mean*_ SON (average temperature)	PG	15.2 ± 2.9	**0.11**	0.09* (5.1**)
	RI	14.1 ± 2.5	0.06	0.08* (2.7)
	PDR	10.0 ± 2.2	0.05	0.05** (3.9)
*T*_*max*_ March (maximum temperature)	PG	15.3 ± 0.5	**0.14**	0.13* (2.6)
	RI	14.5 ± 0.5	0.10	**0.08** (2.1)
	PDR	9.1 ± 0.4	0.17	0.11** (3.5)
*T*_*mean*_ December (average temperature)	PG	6.1 ± 0.3	**0.09**	0.05 (0.15)
	RI	4.9 ± 0.2	**–0.04**	**–0.05** (–4.2)
	PDR	2.3 ± 0.3	**0.11**	0.012** (4.8)

** *p* < 0.01,** *p* < 0.001

Bold values mean a significance of *p* < 0.05

The yearly trends of the *T*_*mean*_, *T*_*min*_, *T*_*max*_ temperatures present a stable difference between the three sites, in terms of degrees of temperature. Indeed, there is a difference of 6^∘^ on average between the higher and the lower gardens, which is noticeable when looking at the temperature trend over time in the three sites (Fig. 3). This yearly constant difference between the three gardens is mirrored in a constant difference of degrees in their climatologies (Fig. 1(b)). Indeed, in the three sites, whose climate can be classified, using the Köppen climate classification as Mediterranean (Lohmann et al. 1993) with a subcontinental influence, the climatology means of the last 14 years—normal over the last 14 years, see Fig. 1(b)—show that in general the Perugia site experiences higher temperatures, followed by the Rieti one and then by Pian de Rosce. In the three sites, the coldest month is January, while the warmest is July. Moreover, the climatologies of Perugia and Rieti tend to overlap within their standard deviations (Fig. 1(b)), while Pian de Rosce climatology does not. The average temperature in Perugia is 14.6 ^∘^*C* (averaging between 5 and 25^∘^*C*) , in Rieti it is 13.1 ^∘^*C* (averaging between 4 and 23^∘^*C*), and in Pian de Rosce it is 9.1 ^∘^*C* (averaging between 1 and 18^∘^*C*).

This is indeed a local effect due to orography, i.e. the different positioning of gardens within the same territory, with Perugia being at 260 m a.s.l. on a hill in the Umbrian valley, Rieti being higher (at 380 m a.s.l.), but positioned in a basin, and Pian de Rosce in the mountains, just above 1000 m. For the seasonal temperature trends over time, similar results have been found, with a significant warming trend for the average spring and autumn temperature for the three gardens (not shown). There are all positive trends, usually more marked for the lower garden site, the Perugia one, and there is the same difference in terms of the degree of temperature between the three gardens. Thus, despite the climatic effect of residing in the same latitudinal range, the three gardens are under the influence of local temperature variations.

The results for the MK test, generally confirm the one from the linear regression, but it shows more significant trends, confirming that the least square method can sometimes fail because of the presence of outliers.

### Correlation analysis between phenology and temperature

The advancement in time of budburst for plants of both species in the three gardens is mainly significant correlated (in some cases even with *p* < 0.001) with the maximum and the average temperature in March (*T*_*max*_ March) and (*T*_*mean*_ March) for both species, ranging between − 0.50 and − 0.80 (Table 3). The budburst also showed a significant correlation with the November and autumn (September, October, November SON) temperature (*T*_*mean*_ SON). By looking at the trends for the above said temperature indices of *T*_*max*_ March (Fig. 3(d)) and *T*_*mean*_ SON (Fig. 3(b)), it can be seen that there is a positive trend for both, which is matching the advance in DOYs. Especially in the case of *T*_*max*_ March, the greatest increase is seen in the Pian de Rosce site (0.17 ^∘^*Cyr*^− 1^, as reported in Table 2) corresponding to the highest phenophase trend (around 1.7 days/year) seen in both species in Fig. 2.

**Table 3:** Pearson correlations between phenophases and most meaningful indices, for all the three gardens and two species

Species	Phase	*T*_*mean*_	*T*_*max*_	*T*_*min*_	*T*_*mean*_ DJF	*T*_*max*_ DJF	*T*_*mean*_ SON	*T*_*min*_ SON	*T*_*mean*_ Feb	*T*_*min*_ Feb	*T*_*mean*_ March
Perugia											
*Salix acutifolia*	BBCH11	–0.90**	–0.74*	0.92**	–0.91**	–0.88**	–0.75**	–0.65**	–0.78*	–0.82*	–0.73**
	BBCH65	0.78*	0.71*	0.76*	**0.63**	**0.64**	0.89**	0.79*	0.40	0.50	–
	BBCH95	0.43	0.45	0.39	–	–	**0.57**	0.66*	–	–	–
*Salix smithiana*	BBCH11	–0.86**	–0.78*	–0.83**	**–0.60**	**–0.61**	**–0.70**	**–0.58**	–0.42	–0.53	–0.75*
	BBCH65	0.91**	0.85**	0.87**	0.76*	0.79*	0.85*	**0.78**	**0.60**	0.44	–
	BBCH95	0.31	0.25	0.32	–	–	**0.54**	**0.74**	–	–	–
Rieti											
*Salix acutifolia*	BBCH11	–0.76*	–0.44	–0.53	–0.15	–0.22	**–0.66**	**–0.62**	–0.14	–0.19	–0.70*
	BBCH65	–0.83**	**–0.64**	–0.53	**–0.54**	–0.74*	**–0.65**	**–0.68**	–0.72*	–0.73*	–
	BBCH95	–0.12	–0.09	–0.08	–	–	**0.74**	**0.72**	–	–	–
*Salix smithiana*	BBCH11	–0.81*	**–0.64**	–0.47	–0.20	–0.48	**–0.66**	**–0.63**	–0.32	–0.35	**–0.52**
	BBCH65	–0.37	–0.71*	–0.24	–0.45	–0.71*	–0.1	–0.13	–0.77*	–0.75*	–
	BBCH95	0.86**	0.47	**0.69**	–	–	**0.76**	**0.89**	–	–	–
Pian de Rosce											
*Salix acutifolia*	BBCH11	–0.11	–0.01	–0.02	–0.20	–0.15	**–0.71**	–0.32	–0.05	–0.07	–0.72*
	BBCH65	0.84**	0.77*	0.38	0.77*	0.83**	**0.57**	–0.15	**0.69**	**0.64**	–
	BBCH95	0.07	0.27	0.50	–	–	**0.78**	**0.69**	–	–	–
*Salix smithiana*	BBCH11	–0.14	–0.20	–0.15	–0.20		**–0.63**	–0.20	–0.10	–0.04	–0.86**
	BBCH65	0.80*	0.81*	0.17	**0.65**	0.81*	0.37	0.02	0.72*	**0.65**	–
	BBCH95	0.45	**0.53**	0.01	–	–	**0.65**	**0.64**	–	–	–
Species	**Phase**	*T*_*max*_ March	*T*_*mean*_ Oct	*T*_*min*_ Oct	*T*_*mean*_ Nov	*T*_*max*_ Nov	*T*_*min*_ Nov	*T*_*mean*_ Dec	*T*_*max*_ Dec	*T*_*mim*_ Dec	
Perugia											
*Salix acutifolia*	BBCH11	–0.85**	–0.55	–0.47	–0.72*	–0.65**	**–0.72**	–0.46	–0.43	–0.42	
	BBCH65	–	0.54	0.49	0.78*	0.71*	0.84*	0.88*	0.81*	**0.83**	
	BBCH95	–	0.65*	0.68*	0.07	0.17	0.19	–	–	–	
*Salix smithiana*	BBCH11	–0.68*	–0.20	–0.13	–0.76*	–0.83*	–0.68*	**–0.56**	**–0.60**	–0.43	
	BBCH65	–	**0.57**	0.52	**0.83**	**0.84**	**0.78**	0.76*	0.75*	0.66*	
	BBCH95	–	0.80*	0.83*	0.32	0.30	0.35	–	–		
Rieti											
*Salix acutifolia*	BBCH11	–0.78*	–0.24	–0.33	**–0.78**	–0.75*	**–0.76**	0.72*	–0.35	**0.68**	
	BBCH65	–	0.37	–0.48	–0.78*	–0.75*	–0.76*	**0.54**	**–0.84**	**0.76**	
	BBCH95	–	**0.50**	**0.47**	0.18	0.39	0.07	–	–	–	
*Salix smithiana*	BBCH11	–0.72*	–0.31	–0.38	**–0.64**	**–0.66**	**–0.69**	0.69*	–0.41	0.70*	
	BBCH65	–	0.03	0.10	–0.15	–0.11	–0.13	**0.58**	–0.65*	0.65*	
	BBCH95	–	**0.79**	**0.75**	**0.85**	**0.84**	**0.80**	–	–	–	
Pian de Rosce											
*Salix acutifolia*	BBCH11	**–0.69**	–0.31	–0.23	–0.79*	**–0.70**	**–0.66**	–0.10	–0.18	–0.24	
	BBCH65	–	0.47	0.34	0.18	0.36	0.02	**0.75**	0.77*	**0.53**	
	BBCH95	–	**0.68**	**0.69**	**0.59**	**0.68**	0.36	–	–	–	
*Salix smithiana*	BBCH11	–0.83**	0.22	0.20	**–0.68**	–0.71*	–0.47	–0.28	–0.40	–0.42	
	BBCH65	–	0.18	0.07	0.49	0.75*	0.15	0.71*	0.83*	**0.54**	
	BBCH95	–	**0.71**	**0.63**	**0.72**	0.84**	0.43	–	–	–	

* *p* < 0.01,** *p* < 0.001

Significant correlations, *p* < 0.05, are in bold

As regards flowering, from the phenological records analysis for the flowering phase, it has been assessed that both willow species present an advance in terms of days of the year for the Rieti garden, while a delay in the other two. In the three gardens, the advancement and the delay are significantly correlated with different temperature indices. For example, in the garden of Perugia, the highest correlation was obtained for *T*_*mean*_ SON of the previous year and for the average, minimum, and maximum December temperature of the previous year (*T*_*mean*_ Dec, *T*_*min*_ Dec, *T*_*max*_ Dec). In the case of the Rieti garden, the Pearson’s coefficients are higher in the case of the average and minimum temperatures of February (*T*_*mean*_ Feb, *T*_*min*_ Feb), *T*_*mean*_ Dec, *T*_*min*_ Dec, *T*_*max*_ Dec and *T*_*mean*_, whereas for the garden of Pian de Rosce, the highest correlations are with *T*_*max*_ Dec and the winter (December-January-February DJF) maximum temperature *T*_*max*_ DJF.

However, the only index which shows opposite results in the case of Perugia and Pian de Rosce on one hand and in the case of Rieti, on the other hand, is *T*_*mean*_ Dec. Indeed, both willow species present a positive correlation with *T*_*mean*_ Dec in the garden of Rieti, while a negative one in the other two. This confirms a common sensitivity of the plants to December warming or cooling. Indeed, *T*_*mean*_ Dec (Fig. 3(f)) presents an opposite trend between the sites of Perugia and Pian de Rosce and that of Rieti. For the first two sites, the temperature trend is positive (Table 2), indicating December warming, while in the case of the third, the temperature trend is negative, indicating cooling. This difference can also be seen in the impact on the trend for *T*_*mean*_ DJF (not shown), where the Rieti site shows a weakly positive trend compared to that of Perugia and Pian de Rosce. Similarly, it can be said also of *T*_*min*_ Dec.

In the case of senescence, in general, for all three gardens and the two species, a significant correlation was found with *T*_*mean*_ SON and minimum autumn temperature (*T*_*min*_ SON) and with the October average (*T*_*mean*_ Oct) and minimum temperature (*T*_*min*_ Oct). The garden’s of Perugia correlation values are slightly lower for *Salix acutifolia* than for *Salix smithiana* (Table 3). The garden that shows the greatest correlations values is in general that of Rieti, especially for the *Salix smithiana*, compared to *Salix acutifolia*. The plants of Pian de Rosce for both species show a significant correlation with all the autumn temperature indices. For *Salix acutifolia* the highest correlation value is for *T*_*mean*_ SON and for *Salix smithiana* is for *T*_*mean*_ Oct.

## Discussion

This study shows that the trends of advance and delay in phenological phases observed in the three gardens situated in central Italy, for two different willow species, have been influenced by the observed rising of temperatures in all the three sites where these shrubs grow over the last 14 years. These results can be reconciled with similar modification of the plants life cycle that has been observed in different regions over the globe (Ren et al. 2018; Piao et al. 2019) in Europe (Pudas et al. 2008; Menzel et al. 2020), regionally, in other Mediterranean climate zones in Italy (Tomasi et al. 2011; Proietti et al. 2020) and for similar short period record (Du et al. 2014; He et al. 2015). The three gardens, in general, show similar trends for all the phenophases (except for the found divergence in the flowering for the Rieti garden), although with different intensities.

From the correlation analysis between phenophases and temperatures trends, it emerges that despite these differences due to the gardens position, the two species share a common dependence on the same temperatures indices; especially the temperature variability of the months preceding the occurrence of the phenophase, in both species, as already found in the literature (Chmielewski et al. 2004; García-Mozo et al. 2010; Piao et al. 2015; Ren et al. 2018).

Concerning budburst, the found results underline that the advancement of this spring phenological phase is mainly correlated with spring warming, according to previous studies (Menzel et al. 2006; Doi and Katano 2008; Fu et al. 2015; Templ et al. 2017; Gerst et al. 2017), specifically with the March maximum temperature which is in line with other spring temperature indices found to explain the budburst trend (Chmielewski et al. 2004; Vitasse et al. 2018) given its sensibility to the preceding months forcing temperatures.

The other noticeable correlation with the warming of autumn temperatures can be combined with the assessed delay in the autumn phenological phase of senescence. (Heide 2003) and (Fu et al. 2014) found that the effect of the delay in leaf senescence is correlated with the anticipation of the budburst, where warm autumn conditions delay the opportunity for bud dormancy induction, which has been shown experimentally in boreal trees. Indeed, the temperature response of spring phenology to warming has been theorized by (Chuine 2000) as a combined effect of the variations of forcing and chilling unit accumulation and found to be true in many studies (García-Mozo et al. 2010; Pletsers et al. 2015; Zhang et al. 2018). The leaf period of the *Smithiana* and *Acutifolia* species under study has been found to increase by about 20 days in the last 14 years, thus in this context, the increase may have influenced the phenological phase of leaf flushing.

Furthermore, the occurrence of delayed leaf senescence, makes it possible to accumulate a higher carbohydrate content (in particular nonstructural carbohydrates), which is thought to be related to budburst through hormonal control (Charrier and Améglio 2011), given the general increase in the leaf period of the plant (Fu et al. 2014). Probably the delay in the senescent phase of the previous autumn can induce a lower accumulation of Abscisic Acid ABA (which accumulates after leaf fall and starch conversion) and therefore a less deep dormancy phase. This condition could lead to an earlier awakening of the plant with an early budburst considering that the cold requirements of the willows in our study areas for this phase were certainly met in the preceding winter months.

In the case of flowering, the divergent pattern found between the gardens of Perugia and Pian de Rosce and that of Rieti for both species, which has already been found in other parts of the globe (Sherry et al. 2007; Guo et al. 2015), can be attributed above all to the different winter heating trend (Cook et al. 2012). Indeed, the delay, or the advancement of flowering, is linked to the plant’s fulfillment of the chilling requirement. With a warmer winter this demand is not met and the plant delays in showing flowers, while with a colder winter, this phase may be anticipated. From our climate analysis, we found that in Rieti the average temperature in December, in particular, is decreasing (Table 2 and Fig. 3(f)), thus contributing to a less pronounced average winter temperature trend.

Indeed, this phenophase correlates well to the average winter temperature and specifically to the average and minimum temperature in December. In a general context of winter warming, the plant is probably more sensitive to the latter than to the spring forcing before the beginning of the phase (Cook et al. 2012; Fu et al. 2015; Roberts et al. 2015). Therefore, with a colder, rather than warmer, December trend, reflected in a less marked trend for the average winter temperature, the plants are more sensitive to this drop in temperature as also found in (Shen et al. 2019).

From a meteorological point of view, Rieti represents an anomaly, regarding this winter cooling, and in general concerning weaker temperature trends. This anomaly could be linked to an accumulation of cold air that comes from the conformation of the valley in which Rieti is located. The Rieti valley is a basin in the middle of the mountains, and this ensures that the cold air descending from the mountains accumulates and stratifies in the plain below, making them susceptible to cold air drainage and pooling (Daly et al. 2007; Jemmett-Smith et al. 2018). This pooling of cold air is what probably guarantees a cooling of the temperature perceived by the plants especially in winter, which can be seen in December’s average temperature in correlation with flowering.

Usually this phenomenon contributes to altering the climate change impacts on similar territories (Daly et al. 2010; Patsiou et al. 2017) as reported in Table 2, Rieti is the site showing generally lower trends in temperature.

Regarding leaf’s senescence, the existence of different behaviour for the two species in the three gardens and the lack of a clear annual variation found in this study for some gardens (Perugia and Rieti), is in line with what has been found in the northern hemisphere (Chmielewski and Rötzer 2001; Estiarte and Peñuelas 2015; Zhu et al. 2017), where inconsistent shifts in the autumn phenology in response to climate warming, especially unchanged leaf senescence, have been reported (Liu et al. 2016; Delpierre et al. 2018). Few studies have reported delaying senescence (Dragoni and Rahman 2012; Yuan et al. 2020), and in general, the dynamics of the latter have received little attention. Generally, the mild temperature during the first autumnal period, which in some cases may induce also the wrong signal to plants (false spring), promotes the tree leaves maintaining and delays the accumulation of hormones (particularly abscisic acid) that induce senescence and thus dormancy.

In literature, the change in senescence has been linked to the timing of leaf flushing (Fu et al. 2018), to a large number of environmental factors, including temperature, precipitation, frost, and photoperiod (Delpierre et al. 2016). This study shows a control from air temperature on the senescence. From our correlation analysis, the overall relationship for both species in the three gardens and the rate of change of senescence was recognized to be the autumn average and minimum temperatures and the average and minimum temperature in October. These latter correlations were recognized in line with literature (Jeganathan et al. 2014; Gill et al. 2015), especially at low latitudes, where rising of temperature in autumn means that the plants are not affected by the cooling necessary to reach the demand to fall leaves (Keenan and Richardson 2015).

In the Pian de Rosce garden, the correlation between the high variability of autumn temperature and the low variability of the onset of the phenophase, turned out to be significant relating it to the probable mediation of this correlation by the different length of the day, which reflects a difference in the radiation received by the plants (Ford et al. 2017).

The elevation, affecting sunlight distribution patterns, may have a high influence on plant phenological traits. Shortwave solar radiation increases with elevation and often during the day at high elevation the maximum solar radiation may be above the saturation levels for photosynthesis of plants determining a relative advantage, as incident sunlight increases with elevation. On the other hand, transpiration rates at high elevations may be very high, resulting in the closure of stomata and inducing a reduction in photosynthesis that may determine xeromorphic plant species morphological and phenological traits (Gale 2004). The environmental control hypothesis represents the most important mechanism behind the occurrence of leaf senescence, triggered when the unfavorable autumn season comes, as a combination of the decrease of both day length and temperature (Farnsworth 2004). In general, this is true for this study, although a more accurate study could be carried out, containing more impact variables such as incoming radiation, at least for the willow plants in the Perugia garden.

Although the phenological phases respond to the same forcing parameters, there are differences in terms of DOYs between gardens and species. The shift in DOYs is definitely related to altitude modulation, although it is not always present and it appears to be species-dependent. When present, the scaling is most probably related to the temperature decrease, which can be observed in all the temperature trends for the three sites with Perugia being the warmer, followed by Rieti and then Pian de Rosce, due to the increase in geographical elevation according to (Prevéy et al. 2017). This usually leads to a significant linear dependence on the elevation of all the phenophases (Pellerin et al. 2012; Gao et al. 2020). This has been found over the Alpine region and in other studies (Larcher 2010).

The presence of budburst scaling with elevation and the absence of a clear elevation scaling for senescence in our study are in line with what has been documented in the literature in (Vitasse et al. 2009; Asse et al. 2018) for budburst and in (Hwang et al. 2014) for senescence. Furthermore, the difference between species elevation scaling that has been found, is in line with what is found in the literature. Indeed, for some trees, (Richardson et al. 2006) found a significant advance with elevation, while in (Vitasse et al. 2009) some contrasting results were found between different species, and sometimes no trend with elevation was found (Jochner et al. 2012).

## Conclusion

This research represents one of the first analyses of the impact of warming in the IPG network located in Central Italy on deciduous trees of two different species. What is primarily highlighted is the effect that temperature variability has on the annual occurrence of the cycle of plants. The observed warming in Central Italy in the last 14 years, specifically in the three garden sites, played a key role in controlling the phenophases occurrences of salix. Even if a difference in terms of DOYs is present among the two species and the three analyzed gardens, the phenological phases present similar trends over time. This corresponds to a dependency on the observed rising temperatures in the region, and it is particularly evident when it comes to budburst and flowering, while for senescence a high yearly variability has been observed.

On the other hand, this study brings to attention the fact that, apart from general temperature correlations, similar in the three gardens, the particular orographic conformation of the Central Italy territory of the Umbria and the Lazio region in the Rieti area, leads to differences in the annual evolution of the two willow species life. These differences can be seen above all in the altitudinal scaling found in the yearly shift of budburst, raging from 5 to 20 days, between the lower gardens (Perugia and Rieti) and the higher one (Pian de Rosce), but also in the trends divergence presented by the gardens in the case of flowering. We can conclude that even though the three gardens are in the same climatic and latitudinal range, there are local effects that add up as impact factors on plant life in Central Italy. Given that 14 years is too short a period to tell whether the advancement of spring phenophases and a delay of autumn phenophases is an assured climatic variability or a consequence of normal temperature variability, our results help in assessing the relative impacts of warming in different orographical positions in Central Italy. As the observation dataset gets longer, more consistent results can be found and the impact of changing climate on deciduous trees in the region can be modeled. An expansion of this initial study is expected to be done also on the other species present in the gardens, so far not analyzed because they have been present for less than 10 years.

In the region, phenophases remain important bio-climatic indicators that provide essential data on how ecosystems are evolving with climate change and can be crucial for making future climate change projections.

## Footnotes

1 p stands for *p*_*v**a**l**u**e*_, i.e. the statistical significance value for the linear regression results. It is the probability of obtaining test results at least as extreme as the results that are actually observed, under the assumption that the null hypothesis is correct. For the linear regression, the null hypothesis is that the slope of the linear relationship is zero. A significance threshold level of *%*5 is commonly used for the linear regression analysis.

## References

[CR1] Allen M, Antwi-Agyei P, Aragon-Durand F et al (2019) Technical summary: global warming of 1.5^∘^ C. An IPCC Special Report on the impacts of global warming of 1.5^∘^ C above pre-industrial levels. IPCC report

[CR2] Asse D, Chuine I, Vitasse Y (2018). Warmer winters reduce the advance of tree spring phenology induced by warmer springs in the Alps. Agr Forest Meteorol.

[CR3] Badeck FW, Bondeau A, Böttcher K (2004). Responses of spring phenology to climate change. N Phytol.

[CR4] Bajocco S, Ferrara C, Alivernini A (2019). Remotely-sensed phenology of Italian forests: going beyond the species. Int J Appl Earth Obs Geoinf.

[CR5] Cerlini BP, Silvestri L (2019) Validation of a regional agro-meteorological network in Central Italy using ECMWF ERA5 reanalysis. Earth and Space Science Open Archive ESSOAr

[CR6] Cerlini BP, Silvestri L, Saraceni M (2020). Quality control and gap-filling methods applied to hourly temperature observations over central Italy. Meteorol Appl.

[CR7] Charrier G, Améglio T (2011). The timing of leaf fall affects cold acclimation by interactions with air temperature through water and carbohydrate contents. Environ Exp Bot.

[CR8] Chmielewski FM, Rötzer T (2001). Response of tree phenology to climate change across Europe. Agr Forest Meteorol.

[CR9] Chmielewski FM, Müller A, Bruns E (2004). Climate changes and trends in phenology of fruit trees and field crops in Germany, 1961–2000. Agr Forest Meteorol.

[CR10] Chmura DJ (2006). Phenology differs among Norway spruce populations in relation to local variation in altitude of maternal stands in the Beskidy mountains. N For.

[CR11] Chuine I (2000). A united model for budburst of trees. J Theor Biol.

[CR12] Cook BI, Wolkovich EM, Parmesan C (2012). Divergent responses to spring and winter warming drive community level flowering trends. Proc Natl Acad Sci USA.

[CR13] Daly C, Smith JW, Smith JI (2007). High-resolution spatial modeling of daily weather elements for a catchment in the Oregon Cascade Mountains, United States. J Appl Meteorol Climatol.

[CR14] Daly C, Conklin DR, Unsworth MH (2010). Local atmospheric decoupling in complex topography alters climate change impacts. Int J Climatol.

[CR15] Deans J, Harvey F (1995). Phenologies of sixteen European provenances of sessile oak growing in Scotland. Forestry: An Int J Forest Res.

[CR16] Delpierre N, Vitasse Y, Chuine I (2016). Temperate and boreal forest tree phenology: from organ-scale processes to terrestrial ecosystem models. Ann For Sci.

[CR17] Delpierre N, Denéchère R, Liu G, et al. (2018) The within-population variability of budburst and leaf senescence is controlled by temperature. In: Phenology 2018

[CR18] Doi H, Katano I (2008). Phenological timings of leaf budburst with climate change in Japan. Agr Forest Meteorol.

[CR19] Dragoni D, Rahman AF (2012). Trends in fall phenology across the deciduous forests of the Eastern USA. Agr Forest Meteorol.

[CR20] Du J, He Z, Yang J (2014). Detecting the effects of climate change on canopy phenology in coniferous forests in semi-arid mountain regions of China. Int J Remote Sens.

[CR21] Estiarte M, Peñuelas J (2015). Alteration of the phenology of leaf senescence and fall in winter deciduous species by climate change: effects on nutrient proficiency. Glob Chang Biol.

[CR22] Farnsworth E (2004). Hormones and shifting ecology throughout plant development. Ecology.

[CR23] Ford KR, Harrington CA, St Clair JB (2017). Photoperiod cues and patterns of genetic variation limit phenological responses to climate change in warm parts of species’ range: modeling diameter-growth cessation in coast Douglas-fir. Glob Chang Biol.

[CR24] Fraga H, Santos J, Moutinho-Pereira J (2016). Observed trends and climate change projections statistical modelling of grapevine phenology in Portuguese wine regions:. J Agric Sci.

[CR25] Fu YH, Zhao H, Piao S (2015). Declining global warming effects on the phenology of spring leaf unfolding. Nature.

[CR26] Fu YH, Piao S, Delpierre N (2018). Larger temperature response of autumn leaf senescence than spring leaf-out phenology. Glob Chang Biol.

[CR27] Fu YS, Campioli M, Vitasse Y (2014). Variation in leaf flushing date influences autumnal senescence and next year’s flushing date in two temperate tree species. Proc Natl Acad Sci.

[CR28] Gale J (2004). Plants and altitude—revisited. Ann Bot.

[CR29] Gao M, Wang X, Meng F (2020). Three-dimensional change in temperature sensitivity of northern vegetation phenology. Glob Chang Biol.

[CR30] García-Mozo H, Mestre A, Galán C (2010). Phenological trends in southern Spain: a response to climate change. Agr Forest Meteorol.

[CR31] Gerst KL, Rossington NL, Mazer SJ (2017). Phenological responsiveness to climate differs among four species of Quercus in North America. J Ecol.

[CR32] Gill AL, Gallinat AS, Sanders-DeMott R (2015). Changes in autumn senescence in northern hemisphere deciduous trees: a meta-analysis of autumn phenology studies. Ann Bot.

[CR33] Gordo O, Sanz JJ (2010). Impact of climate change on plant phenology in Mediterranean ecosystems. Glob Chang Biol.

[CR34] Guo L, Dai J, Wang M (2015). Responses of spring phenology in temperate zone trees to climate warming: a case study of apricot flowering in China. Agr Forest Meteorol.

[CR35] Gutierrez AP, Ponti L, Cossu Q (2009). Effects of climate warming on olive and olive fly (Bactrocera oleae (Gmelin)) in California and Italy. Clim Change.

[CR36] Hänninen H, Häkkinen R, Hari P (1990). Timing of growth cessation in relation to climatic adaptation of northern woody plants. Tree Physiol.

[CR37] He Z, Du J, Zhao W (2015). Assessing temperature sensitivity of subalpine shrub phenology in semi-arid mountain regions of China. Agr Forest Meteorol.

[CR38] Heide O (2003). High autumn temperature delays spring bud burst in boreal trees, counterbalancing the effect of climatic warming. Tree Physiol.

[CR39] Howe GT, Aitken SN, Neale DB (2003). From genotype to phenotype: unraveling the complexities of cold adaptation in forest trees. Can J Bot.

[CR40] Hussain M, Mahmud I (2019). PyMannKendall: a python package for non parametric Mann Kendall family of trend tests. J Open Source Softw.

[CR41] Hwang T, Band LE, Miniat CF (2014). Divergent phenological response to hydroclimate variability in forested mountain watersheds. Glob Chang Biol.

[CR42] Jeganathan C, Dash J, Atkinson P (2014). Remotely sensed trends in the phenology of northern high latitude terrestrial vegetation, controlling for land cover change and vegetation type. Remote Sens Environ.

[CR43] Jemmett-Smith B, Ross AN, Sheridan P (2018). A short climatological study of cold air pools and drainage flows in small valleys. Weather.

[CR44] Jensen J, Hansen J (2008). Geographical variation in phenology of Quercus petraea (matt.) Liebl and Quercus robur l. oak grown in a greenhouse. Scand J For Res.

[CR45] Jochner SC, Sparks TH, Estrella N (2012). The influence of altitude and urbanisation on trends and mean dates in phenology (1980–2009). Int J Biometeorol.

[CR46] Keenan TF, Richardson AD (2015). The timing of autumn senescence is affected by the timing of spring phenology: implications for predictive models. Glob Chang Biol.

[CR47] Kendall M (1975). Rank correlation measures.

[CR48] Klein Tank A, Zwiers F, Zhang X (2009). Guidelines on analysis of extremes in a changing climate in support of informed decisions for adaptation.

[CR49] Körner C (2003). Alpine plant life: functional plant ecology of high mountain ecosystems.

[CR50] Larcher W (2010) Altitudinal variation in flowering time of Lilac (Syringa vulgaris L.) in the Alps in relation to temperatures. Sitzungsberichte und Anzeiger der mathematisch-naturwissenschaftlichen Klasse 1:SBI–3–SBI–18

[CR51] Lee PH, Huang YM, Chiou WL (2018) Fern phenology. In: Current Advances in Fern Research. Springer, pp 381–399

[CR52] Liu Q, Fu YH, Zhu Z (2016). Delayed autumn phenology in the Northern Hemisphere is related to change in both climate and spring phenology. Glob Chang Biol.

[CR53] Lohmann U, Sausen R, Bengtsson L (1993). The Köppen climate classification as a diagnostic tool for general circulation models. Climate Res.

[CR54] Mann HB (1945). Nonparametric tests against trend. Econometrica: J Econ Soc.

[CR55] Menzel A, Sparks TH, Estrella N (2006). European phenological response to climate change matches the warming pattern. Glob Chang Biol.

[CR56] Menzel A, Yuan Y, Matiu M (2020). Climate change fingerprints in recent European plant phenology. Glob Chang Biol.

[CR57] Mimura M, Aitken S (2007). Adaptive gradients and isolation-by-distance with postglacial migration in Picea sitchensis. Heredity.

[CR58] Ohsawa T, Ide Y (2008). Global patterns of genetic variation in plant species along vertical and horizontal gradients on mountains. Glob Ecol Biogeogr.

[CR59] Oleksyn J, Modrzỳnski J, Tjoelker M (1998). Growth and physiology of Picea abies populations from elevational transects: common garden evidence for altitudinal ecotypes and cold adaptation. Funct Ecol.

[CR60] Orlandi F, Garcia-Mozo H, Dhiab AB (2014). Olive tree phenology and climate variations in the Mediterranean area over the last two decades. Theor Appl Climatol.

[CR61] Orlandi F, Ruga L, Bonofiglio T (2016). Plant phenological observations in rural and industrial Central Italy areas. Environ Monit Assess.

[CR62] Orlandi F, Ruga L, Fornaciari M (2020). Willow phenological modelling at different altitudes in Central Italy. Environ Monit Assess.

[CR63] Pachauri RK, Allen MR, Barros VR et al (2014) Climate change 2014: synthesis report. Ipcc

[CR64] Patsiou TS, Conti E, Theodoridis S (2017). The contribution of cold air pooling to the distribution of a rare and endemic plant of the Alps. Plant Ecol Divers.

[CR65] Pellerin M, Delestrade A, Mathieu G (2012). Spring tree phenology in the Alps: effects of air temperature, altitude and local topography. Eur J For Res.

[CR66] Piao S, Tan J, Chen A (2015). Leaf onset in the northern hemisphere triggered by daytime temperature. Nat Commun.

[CR67] Piao S, Liu Q, Chen A (2019). Plant phenology and global climate change: current progresses and challenges. Glob Chang Biol.

[CR68] Pletsers A, Caffarra A, Kelleher CT (2015). Chilling temperature and photoperiod influence the timing of bud burst in juvenile Betula pubescens Ehrh. and Populus tremula L. trees. Ann For Sci.

[CR69] Prevéy J, Vellend M, Rüger N (2017). Greater temperature sensitivity of plant phenology at colder sites: implications for convergence across northern latitudes. Glob Chang Biol.

[CR70] Proietti R, Antonucci S, Monteverdi MC (2020). Monitoring spring phenology in Mediterranean beech populations through in situ observation and Synthetic Aperture Radar methods. Remote Sens Environ.

[CR71] Pudas E, Leppälä M, Tolvanen A (2008). Trends in phenology of Betula pubescens across the boreal zone in Finland. Int J Biometeorol.

[CR72] Rafferty NE, Diez JM, Bertelsen CD (2020). Changing climate drives divergent and nonlinear shifts in flowering phenology across elevations. Curr Biol.

[CR73] Razali NM, Wah YB (2011). Power comparisons of Shapiro-Wilk, Kolmogorov-Smirnov, Lilliefors and Anderson-Darling tests. J Stat Model Anal.

[CR74] Ren S, Yi S, Peichl M (2018). Diverse responses of vegetation phenology to climate change in different grasslands in Inner Mongolia during 2000–2016. Remote Sens.

[CR75] Richardson AD, Bailey AS, Denny EG (2006). Phenology of a northern hardwood forest canopy. Glob Chang Biol.

[CR76] Richardson AD, Keenan TF, Migliavacca M (2013). Climate change, phenology, and phenological control of vegetation feedbacks to the climate system. Agr Forest Meteorol.

[CR77] Roberts AM, Tansey C, Smithers RJ (2015). Predicting a change in the order of spring phenology in temperate forests. Glob Chang Biol.

[CR78] Ruml M, Korać N, Vujadinović M (2016). Response of grapevine phenology to recent temperature change and variability in the wine-producing area of Sremski Karlovci, Serbia. J Agric Sci.

[CR79] Saska MM, Kuzovkina YA (2010). Phenological stages of willow (salix). Ann Appl Biol.

[CR80] Seyednasrollah B, Bowling DR, Cheng R et al (2020) Seasonal variation in the canopy color of temperate evergreen conifer forests. New Phytologist 10.1111/nph.17046 PMC7898516 33118171

[CR81] Shen X, Liu B, Xue Z (2019). Spatiotemporal variation in vegetation spring phenology and its response to climate change in freshwater marshes of Northeast China. Sci Total Environ.

[CR82] Sherry RA, Zhou X, Gu S (2007). Divergence of reproductive phenology under climate warming. Proc Natl Acad Sci USA.

[CR83] Templ B, Templ M, Filzmoser P (2017). Phenological patterns of flowering across biogeographical regions of Europe. Int J Biometeorol.

[CR84] Todisco F, Vergni L (2008). Climatic changes in Central Italy and their potential effects on corn water consumption. Agr Forest Meteorol.

[CR85] Tomasi D, Jones GV, Giust M (2011). Grapevine phenology and climate change: relationships and trends in the Veneto Region of Italy for 1964-2009. Am J Enol Vitic.

[CR86] Visser ME, Both C (2005). Shifts in phenology due to global climate change: the need for a yardstick. Proc R Soc B Biol Sci.

[CR87] Vitasse Y, Porté AJ, Kremer A (2009). Responses of canopy duration to temperature changes in four temperate tree species: relative contributions of spring and autumn leaf phenology. Oecologia.

[CR88] Vitasse Y, Signarbieux C, Fu YH (2018). Global warming leads to more uniform spring phenology across elevations. Proc Natl Acad Sci.

[CR89] White MA, Thornton PE, Running SW (1997). A continental phenology model for monitoring vegetation responses to interannual climatic variability. Global Biogeochem Cycles.

[CR90] Wolf AA, Zavaleta ES, Selmants PC (2017). Flowering phenology shifts in response to biodiversity loss. Proc Natl Acad Sci.

[CR91] Worrall J (1983). Temperature–bud-burst relationships in amabilis and subalpine fir provenance tests replicated at different elevations. Silvae Genet.

[CR92] Yuan M, Zhao L, Lin A (2020). How do climatic and non-climatic factors contribute to the dynamics of vegetation autumn phenology in the Yellow River Basin, China?. Ecol Indic.

[CR93] Yue S, Wang C (2004). The Mann-Kendall test modified by effective sample size to detect trend in serially correlated hydrological series. Water Resour Manag.

[CR94] Zhang Q, Kong D, Shi P (2018). Vegetation phenology on the Qinghai-Tibetan Plateau and its response to climate change (1982–2013). Agr Forest Meteorol.

[CR95] Zhang X, Tarpley D, Sullivan J T (2007) Diverse responses of vegetation phenology to a warming climate. Geophys Res Lett 34(19)

[CR96] Zhang X, Alexander L, Hegerl GC (2011). Indices for monitoring changes in extremes based on daily temperature and precipitation data. Wiley Interdiscip Rev Clim Chang.

[CR97] Zheng J, Ge Q, Hao Z (2006). Spring phenophases in recent decades over eastern China and its possible link to climate changes. Clim Change.

[CR98] Zhu W, Jiang N, Chen G (2017). Divergent shifts and responses of plant autumn phenology to climate change on the Qinghai-Tibetan Plateau. Agr Forest Meteorol.

[CR99] Ziello C, Estrella N, Kostova M (2009). Influence of altitude on phenology of selected plant species in the Alpine region (1971-2000). Climate Res.

